# Mildly elevated liver lipid content is characterised by reduced insulin sensitivity

**DOI:** 10.1016/j.jhepr.2025.101535

**Published:** 2025-08-06

**Authors:** Nelli Tuomola, Eleni Rebelos, Aino Latva-Rasku, Marco Bucci, Heidi Immonen, Virva Saunavaara, Saara Laine, Tanja Sjöros, Taru Garthwaite, Juho R.H. Raiko, Lilian Fernandes Silva, Kirsi A. Virtanen, Jarna C. Hannukainen, Mika Ala-Korpela, Kari K. Kalliokoski, Ilkka H.A. Heinonen, Pirjo Nuutila, Miikka-Juhani Honka

**Affiliations:** 1 Turku PET Centre, University of Turku, Turku, Finland; 2 Turku University Hospital, Wellbeing Services County of Southwest Finland, Turku, Finland; 3 Department of Endocrinology, Turku University Hospital, Wellbeing Services County of Southwest Finland, Turku, Finland; 4 Institute of Clinical Physiology, National Research Council (CNR), Pisa, Italy; 5 Department of Clinical and Experimental Medicine, University of Pisa, Pisa, Italy; 6 Division of Clinical Geriatrics, Department of Neurobiology, Care Sciences and Society, Center for Alzheimer Research, Karolinska Institutet, Stockholm, Sweden; 7 Theme Inflammation and Aging, Karolinska University Hospital, Stockholm, Sweden; 8 Turku PET Centre, Åbo Akademi University, Turku, Finland; 9 Division of Medical Imaging, Department of Medical Physics, Turku University Hospital, Wellbeing Services County of Southwest Finland, Turku, Finland; 10 Institute of Clinical Medicine, Internal Medicine, University of Eastern Finland, Kuopio, Finland; 11 Systems Epidemiology, Research Unit of Population Health, Faculty of Medicine, University of Oulu, Oulu, Finland; 12 Biocenter Oulu, Oulu, Finland; 13 NMR Metabolomics Laboratory, School of Pharmacy, Faculty of Health Sciences, University of Eastern Finland, Kuopio, Finland; 14 Division of Information Science, Nara Institute of Science and Technology, Ikoma, Nara, Japan

**Keywords:** Positron emission tomography, Liver fat content, Insulin resistance, Endogenous glucose production

## Abstract

**Background & Aims:**

Recently, a new cut-off of ≤1.85% was proposed for normal liver lipid content based on a large population trial. In that study, people having liver lipid between 1.86% and the currently used magnetic resonance spectroscopy-specific upper limit of 5.56% had lower insulin sensitivity (higher homeostatic model assessment for insulin resistance [HOMA-IR]) when compared with people with ≤1.85% of liver lipid. We aimed to build upon these findings by evaluating differences in tissue-specific insulin sensitivity between individuals having low (LL; ≤1.85%) or mildly elevated (MEL; >1.85% and ≤5.56%) liver lipids.

**Methods:**

Combining data from previous studies, 202 White European individuals without diabetes were included in this cross-sectional study. Liver lipids were measured with magnetic resonance spectroscopy. Endogenous glucose production (EGP; N = 96) was measured by hyperinsulinaemic–euglycaemic clamp combined with [^18^F]fluorodeoxyglucose positron emission tomography, and adipose tissue insulin resistance by the product of fasting free fatty acids and insulin (N = 197). Serum metabolites were measured using nuclear magnetic resonance metabolomics (N = 152).

**Results:**

The MEL group had higher EGP during hyperinsulinemia (2.7 [-0.4; 7.5] *vs.* -0.2 [-4.3; 5.3] μmol/kg/min, *p* = 0.041) and higher adipose tissue insulin resistance at fasting (28.4 [16.6; 37.5] *vs.* 17.6 [9.6; 26.9] pmol/L × mmol/L, *p* = 0.037) compared with the LL group. In addition, serum triglycerides and branched-chain amino acids were elevated (false discovery rate <0.05) compared with the LL group.

**Conclusions:**

People with MEL had lower hepatic and adipose tissue insulin sensitivity and adverse changes in metabolites when compared with people with LL. These findings support a lower upper limit for normal liver lipids in White Europeans. In addition, the data indicate that impaired suppression of EGP during hyperinsulinaemia and insulin resistance of lipolysis are early features in the cascade of systemic insulin resistance.

**Impact and implications:**

It has long been known that a substantially increased liver lipid content is connected to an increase in cardiovascular risk factors. From the perspective of both researchers and clinicians, it is important to know that even slightly elevated liver lipid content is associated with many adverse metabolic changes. Further research is needed to confirm if intervening early in the development of fatty liver with lifestyle intervention and, if necessary, drug treatment at an early stage, provide benefit for the prevention of metabolic diseases in the future.

**Clinical Trials Registration:**

The study has been registered at ClinicalTrials.gov (NCT03310502).

## Highlights

• The suppression of endogenous glucose production and lipolysis by insulin are impaired at 1.86–5.56% liver lipid content.
• A slightly increased liver lipid content is associated with metabolic risk markers for MASLD, cardiovascular disease, or type 2 diabetes.
• These changes suggest that the upper limit for normal liver lipid content in White European individuals should be <5.56%.

## Graphical abstract

**Figure:**
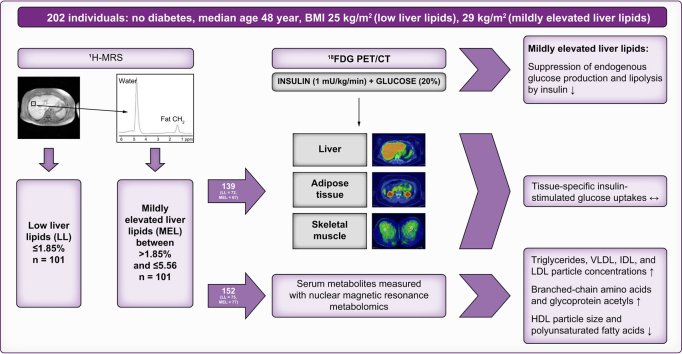

## Introduction

As obesity rates rise globally, the prevalence of metabolic dysfunction-associated steatotic liver disease^1^ (MASLD, previously known as non-alcoholic fatty liver disease [NAFLD]) also increases. This condition currently affects ∼30% of the global population.^1^

Metabolic syndrome and MASLD are closely connected.^2^ By definition, people with MASLD have at least one cardiometabolic risk factor.^3^ Those with metabolic syndrome have significantly increased liver lipid content and hepatic insulin resistance.^2^ MASLD is a major predisposing factor for type 2 diabetes (T2D) and cardiovascular disease.^1^^,^^2^ MASLD can also develop into metabolic dysfunction-associated steatohepatitis (MASH, formerly known as non-alcoholic steatohepatitis [NASH]) and further into cirrhosis and hepatocellular carcinoma.[1], [2], [3]

^1^H magnetic resonance spectroscopy (^1^H-MRS) and magnetic resonance imaging (MRI) proton density fat fraction are standard non-invasive methods to measure hepatic triglyceride content (HTG)^3^ and have partly replaced liver biopsy, which is considered the gold standard for measuring steatosis. It is important to note that for the measurement of HTG, the ^1^H-MRS/MRI measurement of proton density fat fraction and histological evaluation of a liver biopsy provide fundamentally different information. The former is calculated by a measurement of total proportions of hepatic triglycerides and water based on their proton density within the volume of interest and correlates closely with the actual hepatic triglyceride content.^4^ In contrast, during biopsy assessment, a pathologist visually evaluates the proportion of hepatocytes containing macrovesicular lipid droplets on a histological slide. Thus, histological assessment tends to underestimate HTG at the lower range (steatosis grade 0), as small lipid concentrations are not visible as droplets to the pathologist. Conversely, at the higher range of HTG (grades ≥1), histological assessment tends to overestimate HTG, as it accounts for the proportion of hepatocytes containing macrovesicular lipid droplets but does not consider droplet size.^5^

Recently, Petersen and colleagues^6^ proposed a HTG cut-off value of ≤1.85% for normal liver lipid content, based on a large study of approximately 1,500 individuals. This study had a significantly larger sample size and meticulously screened for confounding factors such as excessive alcohol consumption, possible medical conditions, or medications compared with the Dallas Heart Study,^7^ which previously established the HTG cut-off of ≤5.56%. In this cohort that comprised lean, non-Asian participants it was shown that fasting insulin, triglycerides, total cholesterol, LDL cholesterol, and uric acid were increased whereas HDL cholesterol was decreased in people with HTG content >1.85% and ≤5.56% compared to individuals with HTG content ≤1.85%. The higher HTG group was also characterised by increased blood pressure and whole-body insulin resistance (IR) as reflected by increased homeostatic model assessment for IR (HOMA-IR) and lower Matsuda insulin sensitivity index (ISI) after oral glucose tolerance test (OGTT). However, as this study used indirect measurements of insulin sensitivity, it remains unclear which organs are specifically affected in participants with mildly elevated HTG. In addition, although triglycerides and total, LDL, and HDL cholesterol are established risk markers for cardiovascular disease (CVD), the use of serum metabolomics can provide a more detailed picture of the risk for CVD, T2D, and MASLD.[8], [9], [10]

To date, a comprehensive characterisation of the metabolic profiles of individuals with mildly elevated liver lipid (MEL, >1.85% and ≤5.56%) remains lacking. There were two aims in the present study. First, we assessed whether individuals with MEL exhibit worse systemic and tissue-specific insulin sensitivity compared with those with low (LL, ≤1.85%) liver lipid content. Second, by measuring fasting circulating metabolites we evaluated whether MEL participants show potential unfavourable changes in metabolic risk factors compared with LL participants.

## Patients and methods

### Study participants

Our cross-sectional study included 202 (LL = 101, MEL = 101) White European individuals without diabetes from the CMgene metabolic PET study cohort. Of these participants all underwent ^1^H magnetic resonance spectroscopy (^1^H-MRS) of the liver, 139 (LL = 72, MEL = 67) a [^18^F]fluorodeoxyglucose positron emission tomography ([^18^F]FDG-PET) study during insulin clamp, and 152 (LL = 75, MEL = 77) had measurement of serum metabolites with [^1^H] nuclear magnetic resonance ([^1^H]NMR) (Fig. S1). This cohort included participants from previous PET studies (from 2000 to 2019) performed at the Turku PET centre (Turku, Finland). Inclusion criteria for the study was the availability of (1) HTG ≤5.56% according to ^1^H-MRS and (2) [^18^F]FDG-PET study during insulin clamp or serum metabolites with [^1^H]NMR metabolomics (no lipid medication). Individuals with diabetes were excluded from the study. All participants gave written informed consent. The study protocol was approved by the Ethics Committee of the Wellbeing Services County of Southwest Finland and the study has been registered at ClinicalTrials.gov (NCT03310502). All research was conducted in accordance with the Declaration of Helsinki.

### Study design

Participants underwent ^1^H-MRS to assess HTG. [^18^F]FDG-PET during hyperinsulinaemic–euglycaemic clamp (40 mU/m^2^/min) was performed as previously described^11^ to quantify whole-body glucose uptake (M value, whole-body insulin sensitivity), endogenous glucose production (EGP) as well as glucose uptake (GU) in the liver, skeletal muscle, and abdominal subcutaneous adipose tissue (ASAT). The regions scanned and timing when each region was scanned relative to the clamp start varied according to the original PET research protocols (Fig. S2).

All participants underwent anthropometric measurements (height, body weight, waist circumference, and blood pressure). Glucose and insulin were measured in fasting plasma samples to quantify insulin resistance using the HOMA-IR model.^12^ A standard (75 g) OGTT was performed to assess whole-body insulin sensitivity using the simplified Matsuda ISI (calculated as 10,000/sqrt[glucose_0 min_ × insulin_0 min_ × glucose_120 min_ × insulin_120 min_]).^13^ First phase insulin secretion was calculated as insulin_AUC 0–30 min_/glucose_AUC 0–30 min_ (time points 0 and 30 min) and total/second phase as insulin_AUC 0–120 min_/glucose_AUC 0–120 min_ (time points 0, 60, and 120 min) during the OGTT.^14^ Adipose tissue insulin resistance (Adipo-IR) was assessed at fasting and during the hyperinsulinaemic–euglycaemic clamp, calculated as the product of plasma insulin and serum free fatty acid concentrations.^15^

### Measurement of liver triglyceride content and visceral fat mass

^1^H-MRS was performed using a Philips Intera 1.5 T scanner (Philips Medical Systems, Best, The Netherlands; 171 participants), a Philips 3 T system (Ingenuity TF PET/MR; 29 participants), or Siemens Magnetom Skyra fit 3T MRI system (Siemens Healthcare, Erlangen, Germany; two participants). A single voxel was positioned in the liver parenchyma avoiding large vessels to obtain the ^1^H-MRS spectra for the measurement of HTG.^16^^,^^17^ HTG was analysed from the ^1^H-MRS spectra using LCModel (http://s-provencher.com/lcmodel.shtml).

Abdominal MRI was performed during the same session as ^1^H-MRS. Total visceral adipose tissue (VAT) mass was determined either using the prediction formulas developed by Abate *et al.*^18^ based on the amount of intraperitoneal and retroperitoneal fat at the lumbar 2 and 3 vertebrae, or by segmentation of the whole VAT volume.^19^

### PET studies

PET studies were performed after an overnight fast. Participants were instructed not to consume alcohol or caffeine for 12 h and to avoid strenuous physical activity 24 h before the study. The clamp study was performed as previously described.^11^ The rates of whole-body GU (M value) were calculated from steady state and reported as the average of three 20-min intervals, starting after reaching a plasma glucose level of 5 mmol/L.

PET scanners ECAT 931/08 (Siemens Molecular Imaging, Inc., Knoxville, TN, USA), GE Advance, PET/CT Discovery VCT, and PET/CT Discovery 690 (General Electric Medical Systems, Milwaukee, WI, USA) were used. The scanners were cross-calibrated against the same VDC-404 Dose calibrator (COMECER Netherlands, Joure, The Netherlands) to ensure the consistency of the results. All data obtained were corrected for dead time, decay, and measured photon attenuation.

Tissue activity was measured from the images using Carimas software.^20^ Tissue-specific GUs were measured by drawing the regions of interest (ROI) to quadriceps femoris, right lobe of the liver, and abdominal subcutaneous adipose tissue. MRI or CT images were used as references for outlining the regions. The rates of tissue-specific GU were calculated by using the Gjedde-Patlak plot to quantify the fractional phosphorylation rate (Ki) for [^18^F]FDG.^21^ The rates of GU were calculated by multiplying Ki by the plasma glucose concentration and dividing by the tissue density and a lumped constant. A lumped constant corrects for the differences in transportation and phosphorylation of [^18^F]FDG and glucose. Lumped constant values of 1.2 for skeletal muscle, 1.0 for liver, and 1.14 for adipose tissue were used.^11^

### Measurement of endogenous glucose production and glucose rate of disappearance

EGP during the clamp study was measured using a previously validated method.^22^ In brief, EGP was calculated by subtracting the glucose infusion rate from the glucose rate of disappearance (Rd). Rd was obtained from [^18^F]FDG clearance adjusted with [^18^F]FDG lost to urine: Rd = $\frac{FDG\;dose-FDG\;in\;urine}{AUC\;of\;plasma\;FDG}$ × average plasma glucose.^22^

### Biochemical analyses

Plasma glucose during the euglycaemic hyperinsulinaemic clamp was determined in duplicate by the glucose oxidase method (Analox GM9, Analox Instruments, London, UK). Plasma insulin concentration, determined every 30 min during the clamp and from the OGTT, was measured by a double-antibody radioimmunoassay (RIA; Phadeseph Insulin RIA kit, Pharmacia & Upjohn, Uppsala, Sweden), a time-resolved immunofluorometric assay (TR-IFMA, Autodelfia, Wallac, PerkinElmer, Turku, Finland), or electrochemiluminometric immunoassay (ECLIA; Modular E170 or Cobas e601, Roche Diagnostics GmbH, Mannheim, Germany). HbA1c was measured using HPLC (Variant II Turbo; Bio-Rad, Hercules, CA, USA) or photometric immunoturbidimetric method (Tina-quant Hemoglobin A1c Gen 3, Cobas c501, Roche Diagnostics GmbH). Plasma glucose for the OGTT measurements was measured with a photometric, enzymatic method (Modular P800 or Cobas c702, Roche Diagnostics GmbH).

### Genotyping

DNA was isolated from the whole blood. Genotyping was done using the Illumina OmniExpress BeadChip at the Institute for Molecular Medicine (Helsinki, Finland). We used rs4823173 as a proxy for *PNPLA3* (patatin-like phospholipase domain-containing 3) liver steatosis risk variant rs738409 (D′ 1.0, R2 0.965) and rs10401969 as a proxy for *TM6SF2* (transmembrane 6 superfamily member 2) rs58542926 (D′ 1.0, R2 1.0). Gene variants were measured from 93 participants.

### Serum metabolomics

A high-throughput serum [^1^H]NMR metabolomics platform was used to quantify fasting serum metabolite measures from 152 participants at the University of Eastern Finland (Kuopio, Finland) and Nightingale Health Plc (Helsinki, Finland). This method allows simultaneous quantification of lipids, lipoprotein subclass distributions, fatty acids, as well as other low-molecular weight metabolites, such as some amino acids.^9^ Individuals with lipid medication were excluded from this analysis.

### Statistical analysis

Differences between the LL group (HTG ≤1.85%) and MEL (HTG between >1.85% and ≤5.56%) in basic clinical and laboratory measures, and tissue GU, EGP, and M value, were compared using multiple linear regression taking into account sex, BMI, age, and timing of the PET scanning relative to the clamp start where appropriate. The low and high VAT mass groups were compared in a similar manner, but the VAT group was used as a regression predictor instead of the HTG group. Variables in the models were transformed to achieve normal distribution/linearize the relationship between variables when needed. Potential impact of different insulin assays on the comparison of Adipo-IR in fasting or clamp or HOMA-IR between the MEL and LL groups were evaluated by including two of the insulin assays as dummy-coded predictor variables to the regression analysis in addition to study group, age, sex, and BMI. The number of women and men and alleles of *PNPLA*3-and *TM6SF2* gene risk variants between the LL and MEL groups were compared for the descriptive statistics using the chi-squared test. The differences in the NMR metabolomic measures between the LL and MEL groups were compared with logistic regression adjusted for sex, age, and BMI using the forestplotNMR R package. The metabolomic measures were log_2_-transformed before this analysis and are presented in a forestplot where odds ratios and their 95% CIs are shown per one standard deviation difference in the metabolic measures. To account for the high number of tests, false discovery rate (q value) was estimated for each NMR metabolomics measure comparison between the LL and MEL and low and high VAT groups using the R package qvalue (Storey’s method).^23^ The decision to use Storey’s method was based on improved power when the distribution of *p* values is skewed to the right (high effect chance).^24^ (Fig. S3). Statistical testing for the clinical, biochemical, and insulin sensitivity measures were done using IBM SPSS Statistics for Windows (version 27, IBM Corp, Armonk, NY, USA). Strength of evidence was evaluated by using a graded approach: *p* <0.01, strong evidence; 0.01≤ *p* <0.05, moderate evidence; 0.05≤ *p* <0.1, weak evidence; and >0.1 little or no evidence.^25^ The same scale was used for interpreting the false discovery rate (FDR) values in the comparison of NMR metabolomics results. To estimate the required sample size for this study, we used parameters from Petersen *et al.*,^6^ whose report highlighted differences and provided relevant data aligned with our measurements of insulin sensitivity and lipid measurements, including HOMA-IR, Matsuda ISI, LDL cholesterol, and triglycerides. Using the group difference and pooled SD in HOMA-IR (Cohen’s d 0.58) reported in the previous study by Petersen *et al.*, approximately 100 people would have been needed to detect a similar difference in our study, with 80% power and a significance level of 5% (alpha). A similar sized difference in Matsuda ISI (Cohen’s d 0.62), as reported by Petersen *et al.* was calculated to be detectable with 84 participants, in LDL cholesterol (Cohen’s d 0.70) with 68 participants, and in triglycerides (Cohen’s d 0.78) with 54 participants. Based on these estimates, approximately 100 participants were determined to be a sufficient sample size to detect comparable differences in the analysis of insulin sensitivity and lipoprotein/lipid measures. The sample size calculation was performed using G∗Power v.3.1.9.2 (Heinrich Heine University, Düsseldorf, Germany).

## Results

### Clinical characteristics and variables of the groups with LL and MEL

Between-group comparison of general characteristics of the study participants is listed in Table 1. LL (liver lipids ≤1.85%) and MEL (liver lipids >1.85% and ≤5.56%) groups were well-matched in terms of age, but as expected the MEL group had higher BMI. There were also more women in the LL (77%) compared with the MEL group (62%). The distribution of fatty liver risk variants of *PNPLA3* and *TM6SF2* genes by cross-tabulation was similar between the LL and MEL groups. In addition, there was little evidence for differences between the two groups in alanine aminotransferase, gamma-glutamyl transferase, mean corpuscular volume, blood pressure, or in waist circumference measurements (Table 1).

**Table 1:** General characteristics of the groups with low and mildly elevated liver lipids.

	N (LL/MEL)	LL	MEL	*p* value
Hepatic triglycerides (%)	101/101	0.9 (0.6; 1.3)	3.2 (2.5; 4.3)	
Age (years)	101/101	48 (40; 60)	48 (41; 58)	0.662
Sex (number of men/women)	101/101	24/77	38/63	0.033
BMI (kg/m^2^)	101/101	25.2 (22.6; 28.6)	29.4 (25.3; 33.5)	1.0×10^-7^
Waist (cm)	99/96	91 (76; 100)	100 (90; 108)	0.190
Systolic blood pressure (mmHg)	96/96	131 (119; 145)	132 (120;149)	0.653
Diastolic blood pressure (mmHg)	96/96	80 (73; 88)	86 (78; 92)	0.179
Alanine aminotransferase (U/L)	98/93	19 (15; 24)	23 (16; 30)	0.534
Gamma-glutamyl transferase (U/L)	97/93	17 (13; 23)	21 (14; 29)	0.856
Mean corpuscular volume (fl)	97/91	90 (87; 92)	90 (87; 92)	0.696
Number of participants with 0/1/2-3 *TM6SF2* and/or *PNPLA3* risk alleles	44/49	26/17/1	28/19/2	0.821

**Insulin sensitivity at fasting**				
HOMA-IR	101/101	1.31 (0.91; 1.96)	1.79 (1.26; 2.72)	0.085
Adipo-IR (pmol/l∗mmol/l)	97/100	17.6 (9.6; 26.9)	28.4 (16.6; 37.5)	0.037

**Insulin sensitivity and secretion during OGTT**				
Insulin_AUC0-30min_/glucose_AUC0-30min_ (pmol/mmol)	55/53	16.9 (12.7; 26.1)	24.9 (14.3; 34.0)	0.389
Insulin_AUC0-120min_/glucose_AUC0-120min_ (pmol/mmol)	59/67	22.0 (17.4; 33.6)	32.9 (25.8; 51.9)	0.005
Matsuda ISI	61/68	28.7 (21.3; 41.0)	19.2 (10.6; 27.2)	0.011

**Insulin sensitivity during clamp**				
Adipo-IR (pmol/L × mmol/L)	67/64	15.0 (10.9; 33.8)	30.3 (15.4; 39.6)	0.913

Data represented as median (first quartile; third quartile). The comparisons between LL and MEL are adjusted by age, sex, and BMI using multiple linear regression analysis (the comparisons of age and BMI themselves between the LL and MEL group were not adjusted). The *p* value refers to testing the effect of LL and MEL grouping adjusted for age, sex, and BMI. The number of men and women and number of alleles of *PNPLA3*-and *TM6SF2* risk variants between LL and MEL were compared using the Χ^2^ test. Adipo-IR, adipose tissue insulin resistance index; ISI, insulin sensitivity index; LL, low liver lipids (≤1.85%); MEL, mildly elevated liver lipids (>1.85% and ≤5.56%).

### Insulin sensitivity and secretion

#### Fasting

We found moderate evidence that fasting Adipo-IR was higher in the MEL group compared with the LL group and weak evidence that HOMA-IR was higher in the MEL group (Table 1).

#### Oral glucose tolerance test

We obtained moderate evidence that Matsuda ISI was lower and total/second phase insulin secretion higher in the MEL group. However, there was little evidence for a difference in first phase insulin secretion (Table 1).

#### Hyperinsulinaemic–euglycaemic clamp

**Fig. 1:**
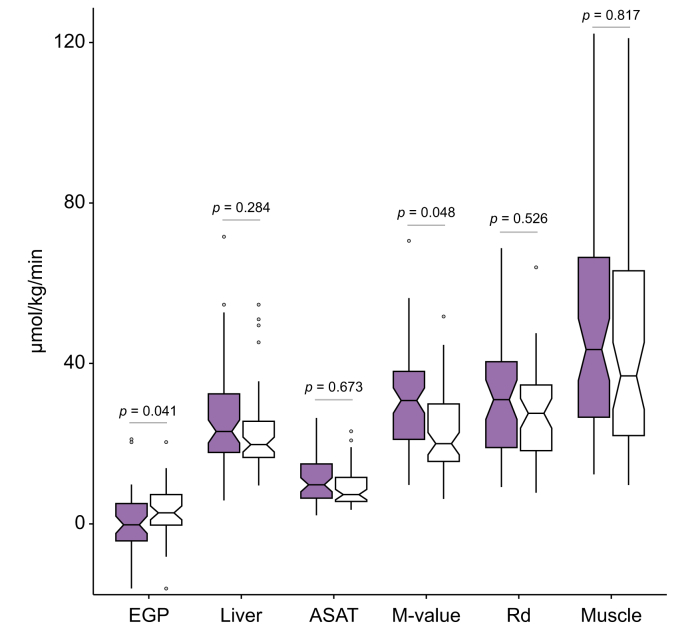
**Insulin sensitivity characteristics of the two study groups measured during hyperinsulinaemic–euglycaemic clamp**. The black bars represent EGP, M value, and GU of the different tissues and organs in the group of low liver lipid content (≤1.85%; LL) and the white bars in the group with mildly elevated liver lipid content (>1.85% and ≤5.56%; MEL). The rates are expressed per kg of body weight for EGP, M value, and Rd and per kg of tissue for liver, ASAT, and muscle GU. The groups were compared using a multivariable model accounting for BMI, age, and sex. In addition, timing of the PET scan was used as a covariate for the comparison of liver, ASAT, and muscle GU between the two groups. Number of cases available for each comparison were: EGP and Rd, n = 96 (LL, n = 47; MEL, n = 49); liver GU, n = 124 (LL, n = 65; MEL, n = 59); ASAT GU, n = 112 (LL, n = 59; MEL, n = 53); M value, n = 139 (LL, n = 72; MEL, n = 67); femoral skeletal muscle GU, n = 127 (LL, n = 66; MEL, n = 61). ASAT, subcutaneous adipose tissue; EGP, endogenous glucose production, GU, glucose uptake; PET, positron emission tomography; Rd, glucose rate of disappearance.

We found moderate evidence for higher EGP and lower M value during hyperinsulinaemic–euglycaemic clamp in MEL compared with the LL group (Fig. 1). However, in a multivariable model, accounting for BMI, age, sex, there was little evidence for a decrease in liver (*p* = 0.258), skeletal muscle (*p* = 0.603), or ASAT GU (*p* = 0.620) in the MEL group. The results regarding tissue-specific GUs remained practically the same when timing of the PET scan was added to the models (Fig. 1). The number of participants with an available M value (N = 139) exceeded those with an EGP measurement (N = 96), as the measurement of [^18^F]FDG urine loss was missing in some studies. However, among those participants who had the EGP measurement, there was little difference in Rd between the groups which align with the tissue-specific GU findings. Adipo-IR during the clamp study did not differ between the two groups (Table 1).

#### Effect of insulin measurement method

Insulin measurements were performed using ECLIA (185 cases), TR-IFMA (11 cases), and double-antibody RIA (Phadeseph; six cases). Thus, there is a possibility that differences in insulin assays may have affected our results. There was no difference in the median fasting insulin measurement between methods (*p* = 0.331), but clamp insulin level was lower among those with Phadeseph measurement (*p* = 3.3×10^-4^ *vs.* ECLIA and *p* = 0.004 *vs.* TR-IFMA). However, including insulin assays as regression predictors had a negligible effect on the comparison of Adipo-IR in fasting or clamp conditions, as well as HOMA-IR between the MEL and LL groups (data not shown). All OGTT results were measured using the ECLIA method.

### Serum metabolomics panel

**Fig. 2:**
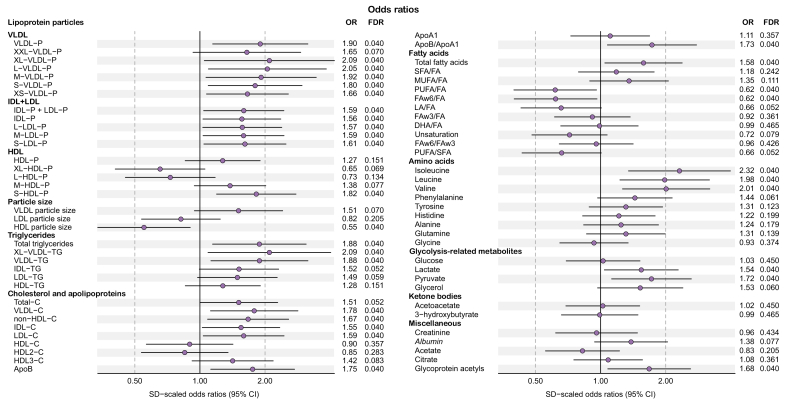
**Cross-sectional associations of metabolic measures with presence of mildly elevated liver lipid content**. Number of participants in the metabolomics analysis was 152 (low fat content, n = 75; mildly elevated fat content, n = 77). The differences in the metabolomic measures between the low and mildly elevated lipid content groups were compared with logistic regression adjusted for sex, age, and BMI. ORs) and their 95% CIs are shown per one standard deviation difference in the metabolic measures and adjusted for sex, age, and BMI. FDR, false discovery rate; OR, odds ratio.

We found moderate evidence for an increase in several serum metabolic measures in the MEL group compared with the LL group at fasting state: serum and VLDL triglyceride; VLDL, IDL, and LDL cholesterol and particle number; apolipoprotein B; total fatty acids; branched-chain amino acids (BCAAs; leucine, isoleucine, and valine); lactate; pyruvate; and glycoprotein acetyls. However, we gained moderate evidence for a decrease in HDL particle size and polyunsaturated (PUFA) and omega-6 fatty acids from total fatty acids in the MEL group (Fig. 2). In addition, the metabolomics panel showed weak evidence for an increase in fasting glycerol level in the MEL group (Fig. 2) and the product of fasting insulin and glycerol, another index of adipose tissue insulin resistance,^26^ was increased (3.75 [2.58; 6.16] *vs.* 1.97 [1.20; 3.21] pmol/l∗mmol/l; *p* = 1.8×10^-4^). This gives further evidence for adipose tissue insulin resistance among participants with MEL. HOMA-IR correlated positively with alanine, glycerol, lactate, and pyruvate which are major substrates of gluconeogenesis (Fig. S4). This suggests that the observed increase in HOMA-IR in MEL is, at least partly, a consequence of increased requirement of insulin to suppress EGP at fasting.

### Effect of visceral fat mass

Besides HTG, fat accumulation into visceral fat depot is known to cause metabolic disturbances. Because HTG correlates with visceral fat mass (Fig. S4), it is possible that the observed differences between MEL/LL are actually a result of visceral fat deposition, rather than HTG. To investigate this possibility, we conducted a separate analysis, dividing the study population in half based on the median visceral fat mass (2.59 kg) and comparing the resulting two groups (174 individuals had the VAT measurement). Similar to the MEL/LL comparison, we found no difference between the groups in skeletal muscle, Rd, or liver GU. However, ASAT GU was lower among individuals with VAT mass above the median, whereas there was no difference in EGP or M value (Fig. S5). The participants with high VAT were more insulin resistant according to HOMA-IR and Adipo-IR at fasting (Table S1). The measurements from OGTT provided moderate evidence for poorer Matsuda ISI and weak evidence for higher first phase insulin secretion in individuals with high VAT mass. In addition, we found moderate evidence for higher Adipo-IR during the clamp study in participants with high fat mass. Interestingly, unlike the MEL/LL comparison, there were few differences in metabolomics measures between the low and high VAT groups when accounting for age, sex, and BMI (Fig. S6). This is compatible with the results of correlation inspection, which showed weaker association between VAT mass and metabolites when compared with HTG and BMI (Fig. S4).

## Discussion

Our study shows that even slightly increased HTG (1.86–5.56%) was associated with unfavourable metabolic changes when compared with lower HTG. People in the MEL group had impaired suppression of EGP and lower M value during euglycaemic hyperinsulinaemia compared with LL group people. This is noteworthy because both of these measures are important contributors to postprandial glycaemic control.^27^^,^^28^ In line with these findings, Matsuda ISI was higher in the LL group than in the MEL group. Matsuda ISI has been found to be a good predictor of worsening of hyperglycaemia, conversion to T2D, and cardiovascular events.^29^ In addition, total/second phase insulin secretion was higher in the MEL group, which is a sign of compensatory insulin secretion in IR.^30^ In the fasting state, individuals with MEL had higher Adipo-IR compared to LL participants, which might have contributed to increased HTG accumulation in this group. Furthermore, the fasting serum NMR metabolomics panel revealed changes in MEL that, alongside traditional risk markers, provide additional insights into the risk of MASLD, CVD or T2D. These include increased levels of serum branched-chain amino acids, apolipoprotein B, and glycoprotein acetyls, as well as a decreased proportion of polyunsaturated fatty acids from total fatty acids, and a lower HDL particle size.[8], [9], [10]^,^^31^

During hyperinsulinemia, insulin enhances GU in skeletal muscle and ASAT by promoting the translocation of the insulin-sensitive glucose transporter GLUT4 (SLC2A4) to the plasma membrane and facilitating glucose phosphorylation via hexokinase II. In the liver, insulin stimulates GU by enhancing glucokinase activity and suppressing glucose production through the inhibition of glycogen phosphorylase, PCK1, and glucose-6-phosphatase activity.^11^^,^^27^^,^^32^ Despite that we observed moderate evidence for a decrease in M value in our study, we saw little evidence for a decline in Rd and GU in the liver, ASAT, and skeletal muscle. Thus, it is possible that participants of the MEL group required less externally infused glucose because of their higher EGP and had a lower M value as a consequence. These results suggest that in the process leading to MASLD, deterioration in the suppression of EGP occurs before a marked decline in insulin’s effect on GLUT4 or hexokinase/glucokinase activity. This interpretation is in line with the previous findings of Bril *et al.*^33^ who found that insulin suppression of EGP deteriorates considerably when HTG increases above 1.5%, whereas Rd showed a more gradual decline with increasing HTG. The results from a sensitivity analysis, in which we split our dataset in half based on VAT mass regardless of HTG, provided additional insight into the pathophysiological processes underlying our findings. In this analysis, EGP did not differ between individuals below or above median VAT mass, whereas ASAT GU was lower among those with higher VAT mass. Additionally, we observed higher Adipo-IR during a clamp study in individuals with higher VAT mass but found no difference when comparing the MEL/LL groups. These findings are plausible, as hepatic glucose production is influenced by local (i.e. intrahepatic) fat accumulation, whereas ASAT GU and Adipo-IR are associated with AT mass.

HTG of 5.56%, previously considered the upper limit of normal HTG, was based on the Dallas Heart Study.^7^ This limit has since been called into question, with recent studies suggesting lowering the upper limit to 3%^34^^,^^35^ and further by Petersen *et al.*^6^ to 1.85%. In the recent study by Petersen *et al.*, participants with HTG between 1.85 and 5.56% had higher HOMA-IR and lower Matsuda ISI as well as increased cardiovascular risk factors compared to people with HTG ≤1.85%. Our findings are in line with the results of Petersen *et al.* Moreover, we characterised further early metabolic derangements in this group of patients, identifying also altered metabolite profile, which have emerged in the recent years as novel markers of CVD,^9^ MASLD,^10^ and T2D.^8^

One important consideration is that in the study by Petersen *et al.*, the findings regarding metabolic alterations were consistent also in lean individuals. As the BMI of the MEL group in our study was mostly >25 kg/m^2^, we were unable to assess whether higher HTG associates with worse insulin sensitivity also in lean individuals. Nevertheless, the ReTUNE study,^36^ which explored the aetiology of T2D, found that HTG among normal-weight patients with T2D was predominantly within the MEL range, whereas matched participants with normal glycaemic control were primarily in the LL range. Interestingly, 70% of these patients with T2D achieved remission lasting 12 months after a weight-loss intervention, while their HTG had fallen to the LL range within the same time span. Although the patients with T2D in the ReTUNE study represented a different population compared with our study, the remission observed after the intervention aligns with the higher insulin sensitivity in the LL group in our study and provides insights into the potential clinical benefits of lowering HTG in patients with T2D.

In another study, Gastaldelli *et al.*^37^ studied 14 non-obese and glucose tolerant individuals, 24 non-obese people with T2D, and 19 people with obesity and T2D and found that impaired EGP suppression during hyperinsulinaemic–euglycaemic clamp correlated with both increased VAT amount and HTG. The increase in VAT amount was predominantly linked to accelerated gluconeogenesis, whereas the increase in HTG was primarily associated with hepatic IR and showed a stronger correlation with diabetes than with obesity. This underscores that the accumulation of HTG, even in lean individuals, significantly increases the risk of hepatic insulin resistance and the development of diabetes. In our study, EGP correlated with HTG content, consistent with the findings of Gastaldelli's group.

We also found that the concentration of serum triglycerides measured by the [^1^H]NMR metabolomics panel was higher in the MEL group compared with the LL group. Elevated triglyceride levels are strongly associated with an increased risk of CVDs^38^ and were recently connected to earlier onset of NAFLD.^39^ Unlike the comparison between the MEL and LL groups, there was no difference in serum triglycerides when the population was divided into high and low VAT mass groups, regardless of HTG. This aligns with the role of hepatic VLDL triglyceride production as a key determinant of circulating triglyceride levels. In fact, previous studies have shown a dramatic increase in hepatic lipogenesis and VLDL triglyceride export when HTG rises from <2 to 5%, whereas at higher HTG levels, increases in lipogenesis and triglyceride export become more gradual.^40^^,^^41^ This means that much of the metabolic derangement in lipid metabolism already occurs below the current upper limit of normal HTG. It is also important to note that the risk associated with increased serum triglycerides is not limited to CVD. Studies using low-grade lipid infusions have shown an induction of IR by mild hypertriglyceridemia^42^ and we have recently demonstrated that higher serum triglycerides at fasting independently associate with poorer glycaemic control during an OGTT.^27^ In addition, a recent Mendelian randomisation study among Europeans suggested that serum triglycerides contribute to insulin resistance.^43^ The mechanism by which higher serum triglycerides could cause insulin resistance is likely increased delivery of fatty acids to insulin-sensitive tissues.^44^ In addition, a considerable proportion of fatty acids are not retained by local tissue, when serum triglycerides are hydrolysed, but released to circulation.^45^ Insulin stimulates triglyceride synthesis and inhibits lipolysis, which reduces the release of fatty acids and glycerol. Reduced insulin sensitivity of adipose tissue therefore leads to higher circulating fatty acid and glycerol concentrations as a result of impaired triglyceride synthesis and antilipolysis in adipocytes.^15^ In our study, the MEL group had poorer suppression of lipolysis at fasting which may have contributed to their elevated HTG accumulation and lower insulin suppression of EGP. There was little difference in Adipo-IR during clamp between the MEL/LL groups but when the study population was divided by VAT mass, the group with higher VAT mass had increased Adipo-IR. This aligns with our [^18^F]FDG-PET data, which showed little differences in ASAT GU between the MEL/LL groups under insulin clamp conditions but lower ASAT GU among individuals with higher VAT when the population was divided according to VAT mass. Based on these findings, higher VAT mass causes adipose tissue insulin resistance independent of HTG and is a likely contributor to the increased HTG in the MEL group.

From the studied NMR metabolic measures, BCAAs showed the strongest association with MEL. However, there was also evidence for increases in VLDL (especially larger) and small HDL particle concentrations, VLDL triglycerides, VLDL and LDL cholesterol, apolipoprotein B, pyruvate, as well as glycoprotein acetyl concentrations. In contrast, HDL particle size and the amount of PUFAs and omega-6 fatty acids from total fatty acids were inversely associated with MEL. A higher PUFA/fatty acid ratio has been found to be associated with reduced risk of coronary artery disease, peripheral artery disease, and T2D, whereas a higher level of glycoprotein acetyls, an inflammatory biomarker, is associated with higher risk.^8^^,^^31^^,^^46^ These results closely resembled those of a large population study conducted by Kaikkonen *et al.*,^10^ which used the same NMR panel to assess predictors of both current and future fatty liver disease, with HTG evaluated via ultrasound. The BCAAs leucine, isoleucine, and valine were elevated in the MEL group. BCAAs are important substrates of protein synthesis and leucine also participates in the regulation of this synthesis by stimulating protein synthesis and reducing protein catabolism, and isoleucine and valine are also substrates for gluconeogenesis.^47^ Intake of leucine and isoleucine induces insulin secretion, but high concentrations of amino acids are associated with inhibition of glucose metabolism and increased risk of T2D.^47^ Genetic studies in human cohorts have provided evidence that insulin resistance increases BCAA concentrations.^48^^,^^49^ In addition, a recent study provided evidence that amino acids are major carbon source of hepatic lipogenesis in obesity.^50^ However, the role of BCAAs in the pathogenesis of T2D is controversial based on genetic studies^48^^,^^51^^,^^52^ and a recent study did not find evidence for an effect of BCAAs on MASLD risk or *vice versa*.^53^ Experimental studies have provided robust evidence for reduced BCAA metabolism in adipose tissue in obesity whereas studies addressing the effect of BCAA intake on insulin resistance have yielded mixed results.^47^ Therefore, it is likely that the increased BCAA levels in the current study are a consequence of their impaired catabolism caused by insulin resistance, but further study is needed to understand whether they contribute to MASLD or diabetes pathogenesis themselves.

It is important to note that while only 5% of lean, healthy individuals had HTG above 1.85% in the study by Petersen *et al.*, the proportion of people exceeding this threshold in the general population would be much higher. Based on a Northeastern German adult general population trial^54^ and a study of consecutive tertiary care centre patients who underwent liver MRI,^55^ the frequency of abnormal liver fat would be 70–80% if the upper limit of the normal range were lowered to 1.85%. This is more than twice as high as the frequency observed with the currently used threshold. Although it is not feasible to screen everyone with MRI or ^1^H-MRS because of high costs and limited availability, the findings of this study and others reviewed here highlight the need to assess whether lowering the cut-off would provide benefits for prevention and treatment in populations with different risk backgrounds.

It is well-established that liver steatosis can also result from common genetic variants, such as PNPLA3-I148M and TM6SF2-E167K. However, fatty liver induced by these variants is not associated with insulin resistance or other features of metabolic syndrome.^2^ Carriers of the gene variants PNPLA3-I148M (21.5% worldwide prevalence^56^) or TM6SF2-E167K (6.8% prevalence^56^) have been found to accumulate PUFAs in the liver, whereas in MASLD, hepatic triglycerides are predominantly saturated or monounsaturated.^57^ Retention of PUFAs in the liver leads to anti-atherogenic changes in the plasma lipid profile,^58^ especially in insulin-resistant individuals, thereby protecting these otherwise high-risk individuals from CVDs but predisposing them to the development of MASH and liver fibrosis. Nevertheless, it is unlikely that our findings are attributable to variations in PNPLA3-I148M or TM6SF2-E167K, as the distribution of these variants was similar between the LL and MEL groups in our study, based on the subset of 93 participants with available genotype data. In addition, the risk allele sum did not appear as a significant predictor when added to the LL/MEL comparisons.

Regrettably, alcohol consumption among study participants was not systemically assessed, either through questionnaires or direct markers of alcohol use, representing a major limitation of our study. However, the commonly used indirect markers of alcohol consumption, gamma-glutamyl transferase or mean corpuscular volume^59^ were not different between the groups. Furthermore, higher alcohol consumption is usually marked by higher serum HDL cholesterol and apolipoprotein A1 concentrations^60^ but we found no difference in their concentrations between the MEL and LL groups. In addition, HDL particle size was notably smaller, and the apolipoprotein B to apolipoprotein A ratio was higher in the MEL group, contrasting with the patterns typically observed with increasing alcohol consumption.^60^ These findings would suggest that the higher HTG in the MEL group was unlikely caused by excessive alcohol consumption. It is important to note that these indirect markers have limited sensitivity,^59^ making it impossible to entirely rule out higher alcohol consumption as a possible contributor to the higher HTG levels in the MEL group. In addition, if alcohol consumption does not account for the increased HTG in the MEL group, an alternative explanation for the heightened fat infiltration must be considered. As mentioned previously, the MEL group exhibited poorer suppression of lipolysis by insulin. We suggest that this impaired lipolysis, combined with the higher BMI of the MEL group, may explain the elevated HTG levels. Furthermore, the use of different MRI scanners in the current study is a potential source of bias for the measurement of HTG. However, HTG values were not different between the groups of individuals with similar age and BMI range studied with either the Philips 1.5 or 3T system (data not shown). Another limitation of the current study is that a measurement of physical fitness was available for less than a third of the participants and we were not sufficiently powered to assess its potential effects on our results. The different insulin assays in subsets of our population could have also affected our results. However, including the insulin assays as factors to the comparison of Adipo-IR or HOMA-IR had negligible effect on our results. Lastly, because of high cost and logistical demands of measuring HTG and tissue-specific GUs using the gold-standard methods, we chose to use previously collected data for this study. This approach yielded somewhat limited sample size regarding PET, EGP, metabolomics, and OGTT measurements and imbalances in BMI and sex between the studied groups. In addition, because of complex logistics of the included studies, the ^1^H-MRS was performed on average 1–2 weeks apart from PET and blood sampling. However, previous studies have reported very good reproducibility between ^1^H-MRS HTG measurements over 1- to 6-month intervals.^61^ Thus, even though all the measurements could not be done on the same day, this is probably not a major limitation for our study. EGP was assessed only during hyperinsulinaemic–euglycaemic clamp but not at fasting state where impaired suppression of EGP is an important contributor of hyperglycaemia which is a limitation of our study.^28^ However, we did measure HOMA-IR, an indirect marker of fasting hepatic insulin resistance, and observed an increase in MEL. This is compatible with the increased gluconeogenic substrate delivery implied by higher fasting Adipo-IR, lactate, pyruvate, and trends for an increase in several glucogenic amino acids. Further, we have recently reported in a cross-sectional setting that higher EGP during a clamp study is a key predictor of poorer glycaemic control during an OGTT.^27^ This finding demonstrates the importance of insulin-mediated EGP suppression in postprandial glycaemic control, previously shown also by others.^28^ Moreover, our findings regarding tissue-specific insulin resistance among the participants with MEL are compatible with the higher HOMA-IR and lower Matsuda ISI observed in the previous study by Petersen *et al.*^6^ In addition, the pattern of higher serum and VLDL triglycerides, VLDL, IDL, and LDL particles, higher apolipoprotein B, total fatty acids, BCAAs, and glycoprotein acetyls and lower HDL particle size, PUFAs, and omega-6 fatty acids in the MEL group was highly similar to that observed among individuals with present fatty liver or at risk for future fatty liver in the considerably larger study from Kaikkonen *et al.*^10^

To conclude, our findings reveal evidence of insulin resistance across multiple organs and adverse metabolic alterations in people with liver lipids between 1.86% and 5.56%, compared with those with liver lipids of 1.85% or lower. In addition, the data indicate that impaired suppression of EGP during hyperinsulinaemia and insulin resistance of lipolysis are early features in the cascade of systemic insulin resistance. These results support the recently proposed reduction of the upper threshold for normal liver lipid content in White European individuals. More research is needed to establish cause-and-effect relationships and to develop effective prevention and treatment strategies for MASLD.

## Abbreviations

[^18^F]FDG, [^18^F]fluorodeoxyglucose; [^1^H]NMR, [^1^H]nuclear magnetic resonance; ^1^H-MRS, ^1^H magnetic resonance spectroscopy; Adipo-IR, adipose tissue insulin resistance; ASAT, abdominal subcutaneous adipose tissue; BCAAs, branched-chain amino acids; CVD, cardiovascular disease; ECLIA, electrochemiluminometric immunoassay; EGP, endogenous glucose production; GGT, gamma-glutamyl transferase; GU, glucose uptake; HOMA-IR, homeostatic model assessment for IR; HTG, hepatic triglyceride content; IR, insulin resistance; LL group, low liver lipid group; MASH, metabolic dysfunction-associated steatohepatitis; MASLD, metabolic dysfunction-associated steatotic liver disease; Matsuda ISI, Matsuda insulin sensitivity index; MEL group, mildly elevated liver lipid group; MRI, magnetic resonance imaging; NAFLD, non-alcoholic fatty liver disease; NASH, non-alcoholic steatohepatitis; OGTT, oral glucose tolerance test; PET, positron emission tomography; PNPLA3 gene, patatin-like phospholipase domain-containing 3 protein gene, PUFA, polyunsaturated fatty acids; Rd, rate of disappearance; RIA, radioimmunoassay; T2D, type 2 diabetes; TM6SF2 gene, transmembrane 6 superfamily member 2 gene; TR-IFMA, time-resolved immunofluorometric assay; VAT, visceral adipose tissue.

## Financial support

The study was conducted at the Center of Excellence in Cardiovascular and Metabolic Diseases, supported by the Research Council of Finland (grant 307402 to PN), the University of Turku, Turku University Hospital, and Åbo Akademi University. NT has received funding from TYKS Foundation, and Turunmaa Duodecim Society. SL has received funding from the Instrumentation Science Foundation, Juho Vainio Foundation, Yrjö Jahnsson Foundation, Turku University Foundation, and the Hospital District of South-West Finland. TS was supported by the Finnish Cultural Foundation, the Finnish Diabetes Research Foundation, Yrjö Jahnson Foundation, Turku University Foundation, TYKS Foundation, and the Hospital District of Southwest Finland. MAK was supported by a research grant from the Sigrid Juselius Foundation, the Finnish Foundation for Cardiovascular Research, and the Research Council of Finland (grant no. 357183). MJH has received funding from the Research Council of Finland (332151), the Finnish Cultural Foundation Varsinais-Suomi regional fund, the Finnish Diabetes Research Foundation, the Finnish-Norwegian Medical Foundation, Jalmari and Rauha Ahokas Foundation, COMEGIR s.r.l., and State Research Funding (Turku University Hospital).

## Authors’ contributions

CMgene study planning: PN, MJH. Performed clinical studies: MB, ALR, HI, SL, TS, TG, KAV, JH, KKK, IHAH. Analysed imaging data: MB, HI, ALR, VS, SL, TS, TG, KAV, MJH. Provided expertise regarding the analysis of MASLD risk variants: LFS. Provided expertise and resources for the NMR metabolomics analyses: MAK. Statistical analysis: MJH. Manuscript draft: NT, MJH. Manuscript review and comments: ER, ALR, MB, HI, VS, SL, TS, TG, JRHR, LFS, KAV, JCH, MAK, KKK, IHAH, PN. Read and approved the final manuscript: all authors.

## Data availability

The data that support the findings of this study are available from the corresponding author upon reasonable request.

## Conflicts of interest

NT has received support for attending meetings from MSD and Accord Healthcare. LFS has received a grant from the Sigrid Juselius Foundation. KAV has received grants from the Novo Nordisk Foundation, Sigrid Juselius Foundation, Finnish Diabetes Research Foundation, Finnish Government Research Funds, Research Council of Finland, Finnish Medical Foundation; consulting fees from Novo Nordisk and Eli Lilly; payment or honoraria from Novo Nordisk, University Hospital of Kuopio, University of Oulu, American Diabetes Association, and European Association for the Study of Diabetes; support for attending meetings or travel from Novo Nordisk, University of Granada, University of Nice, TRR333 BATenergy, University Hospital of Kuopio, and Scandinavian Society of Diabetes; participated on a Data Safety Monitoring Board or Advisory Board for Novo Nordisk and Eli Lilly; has served a board member for the Finnish Association for Obesity- and Metabolic Surgery and President of the Finnish Diabetes Society 2023-2024, Treasurer of the Society 2017-2022, Board member 2011-2024. PN has received grants from the Novo Nordisk Foundation and Minerva Foundation and is currently serving as President of the Finnish Diabetes Research Foundation. The other co-authors reported no potential conflicts of interest relevant to this article. Please refer to the accompanying ICMJE disclosure forms for further details.
