# Supplementary material for: Mildly elevated liver lipid content is characterised by reduced insulin sensitivity
Source: JHEP Rep. 2025 Aug 6;7(11):101535. doi: 10.1016/j.jhepr.2025.101535 (PMC12529492; doi:10.1016/j.jhepr.2025.101535)
Supplement: Multimedia component 1 [file mmc1.pdf]

Mildly elevated liver lipid content is characterised by reduced insulin sensitivity

Nelli Tuomola, Eleni Rebelos, Aino Latva-Rasku, Marco Bucci, Heidi Immonen, Virva Saunavaara, Saara Laine[1], Tanja Sjöros, Taru Garthwaite, Juho R.H. Raiko, Lilian Fernandes Silva, Kirsi A. Virtanen, Jarna C. Hannukainen, Mika Ala-Korpela, Kari K. Kalliokoski, Ilkka H.A. Heinonen, Pirjo Nuutila, Miikka-Juhani Honka

Table of contents

Fig. S1………………..……………………………………………………………………….2

Fig. S2………………..……………………………………………………………………….3

Fig. S3………………..……………………………………………………………………….3

Fig. S4………………..……………………………………………………………………….4

Fig. S5………………..……………………………………………………………………….5

Fig. S6………………..……………………………………………………………………….6

Table S1……………………………………………………………………………………..….7

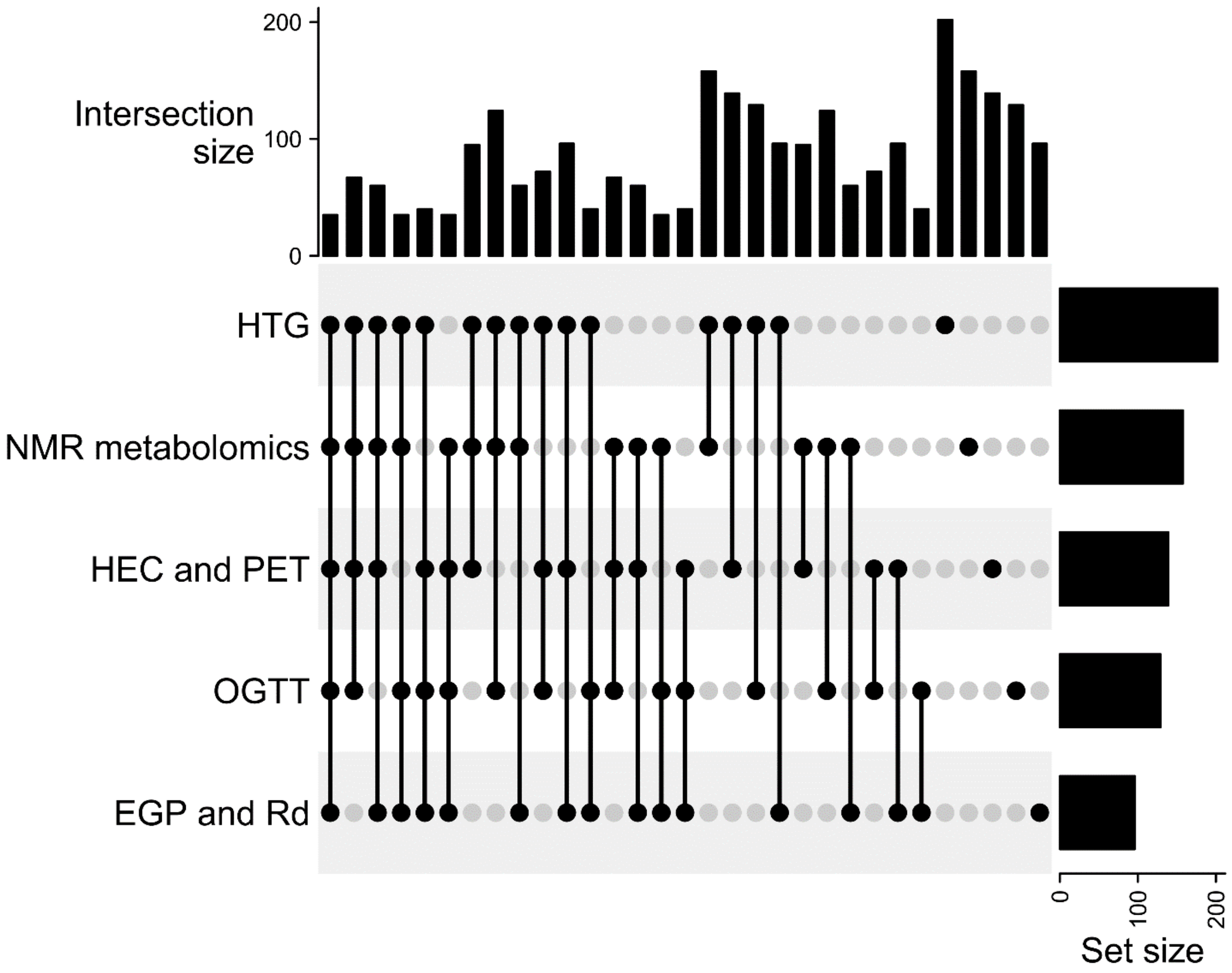

Fig. S1. This intersection plot shows the number of participants with different measurements. The combination of measurements are indicated by the vertically connected dots and intersection size on top shows the number of participants who have those measurements. HTG, hepatic triglycerides; NMR, nuclear magnetic resonance; HEC, hyperinsulinaemic-euglycaemic clamp; PET, positron emission tomography; OGTT, oral glucose tolerance test; EGP, endogenous glucose production; Rd, glucose rate of disappearance.

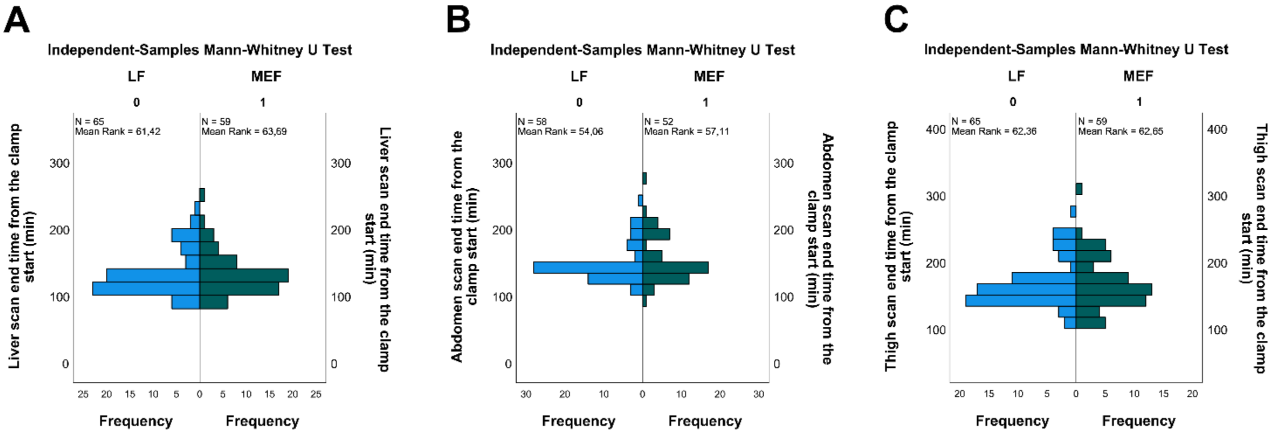

Fig. S2. End time for the liver (A), abdomen (B), and thigh scan (C). The distribution of liver (P = 0.726), abdomen (P = 0.617), or thigh scan (P = 0.964) timings were not different between the groups of low or mildly elevated liver lipid content.

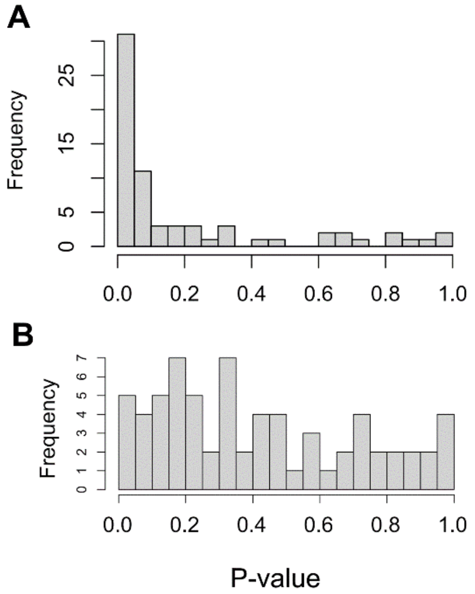

Fig. S3. Histogram of P-values from the comparison of NMR metabolomics measures between groups of low and mildly elevated liver lipid content (A) and between groups of low and high visceral adipose tissue mass (B). The distribution A is typical for a high effect chance.

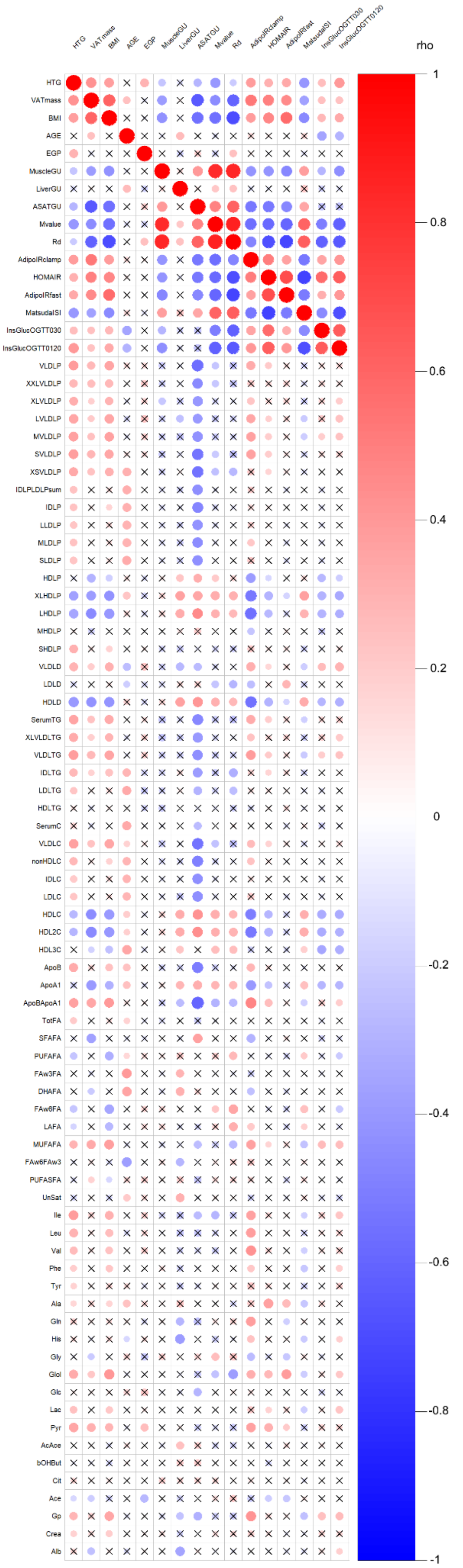

Fig. S4. Spearman correlations between the studied variables. The size and colour intensity of the circles indicate strength of association. Correlations with P > 0.05 are crossed out. HTG, hepatic triglycerides; VATmass, visceral adipose tissue mass; EGP, endogenous glucose production; GU, glucose uptake; ASAT, abdominal subcutaneous adipose tissue; Rd, glucose rate of disappearance; InsGlucOGTT030 and -0120, the ratio of insulin and glucose AUCs from 0 to 30 min and from 0 to 120 min. Lipoprotein particle sizes are described by the following prefixes: XXL, extremely large and chylomicrons; XL, very large; L, large; M, medium; S, small; XS, very small. Lipoprotein particles and their composition are described by the following suffixes: P, particle concentration; D, diameter; L, total lipids; PL, phospholipids; C, cholesterol; CE, cholesteryl esters; FC, free cholesterol; TG, triglycerides. Apo, apolipoprotein; FA, fatty acids; totFA, total fatty acids; SFA, saturated fatty acids; FAw3, omega-3 fatty acids; FAw6, omega-6 fatty acids; DHA, docosahexaenoic acid; LA, linoleic acid; MUFA, monounsaturated fatty acids; PUFA, polyunsaturated fatty acids. Fatty acid ratios are named as fatty acid type followed by FA, e.g. SFAFA, ratio of saturated fatty acids to total fatty acids. UnSat, degree of unsaturation; Ile, isoleucine; Leu, leucine; Val, valine; Phe, phenylalanine; Tyr, tyrosine; Ala, alanine; Gln, glutamine; His, histidine; Gly, glycine; Glol, glycerol; Glc, glucose; Lac, lactate; Pyr, pyruvate; AcAce, acetoacetate; bOHBut, 3-hydroxybutyrate; Cit, citrate; Ace, acetate; Gp, glycoprotein acetylation; Crea, creatinine: Alb, albumin.

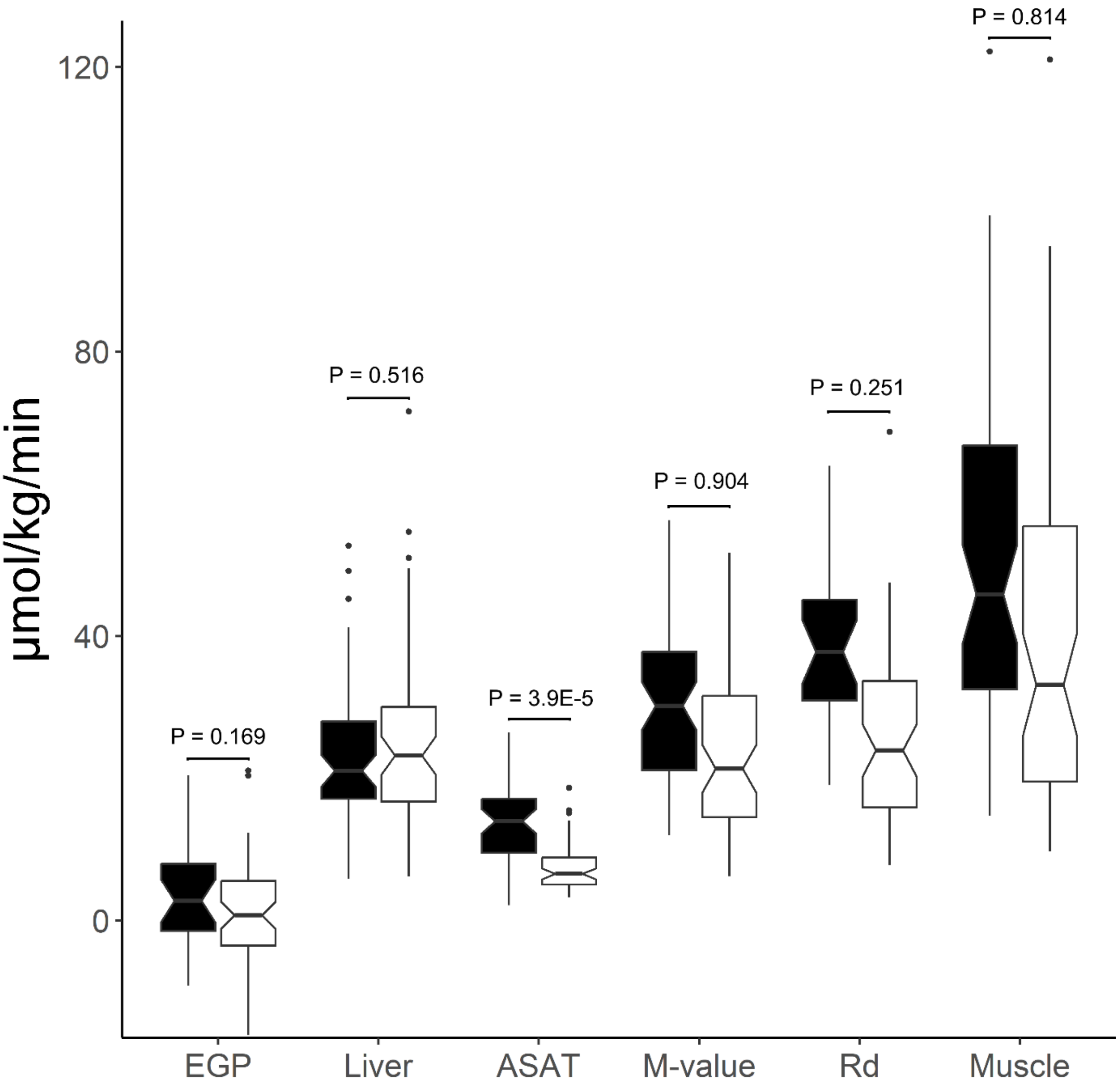

Fig. S5. Insulin sensitivity characteristics of the low and high visceral adipose tissue mass groups measured during hyperinsulinemic euglycemic clamp. EGP = endogenous glucose production, ASAT = subcutaneous adipose tissue, Rd = glucose rate of disappearance. The black bars represent EGP, M value, and glucose uptake of the different tissues and organs in the group of low visceral adipose tissue mass and the white bars in the group with high visceral adipose tissue mass. The groups were compared by using a multivariable model accounting for BMI, age, and sex. In addition, timing of the PET scan was used as a covariate for the comparison of liver, ASAT, and muscle glucose uptake between the two groups. Number of cases available for each comparison were: EGP and Rd, n = 83 (low, n = 25; high, n = 58); liver GU, n = 121 (low, n = 60; high, n = 61); ASAT GU, n = 110 (low, n = 49; high, n = 61); M value, n = 126 (low, n = 62; high, n = 64); femoral skeletal muscle GU, n = 124 (low, n = 62; high, n = 62).

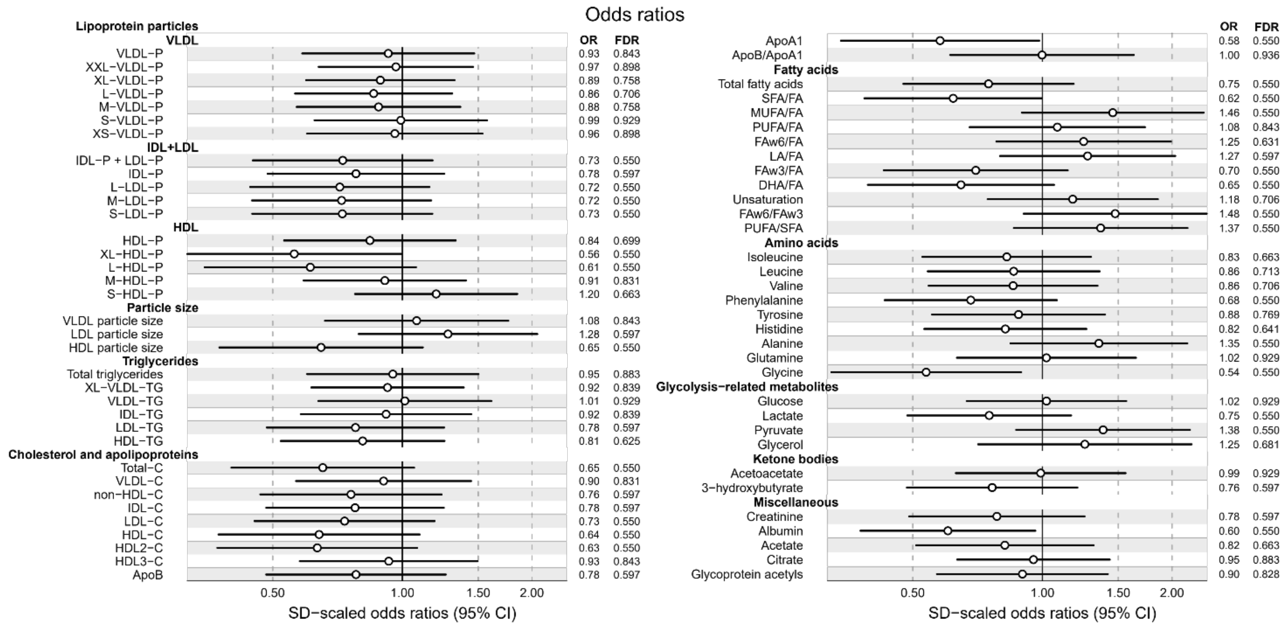

Fig. S6. Cross-sectional associations of metabolic measures with presence of low and high visceral adipose tissue mass. Number of participants in the metabolomics analysis was 132 (low visceral adipose tissue mass, n = 80; high visceral adipose tissue mass, n = 52). The differences in the metabolomic measures between the low and high visceral adipose tissue mass groups were compared with logistic regression adjusted for sex, age, and BMI. Odds ratios (OR) and their 95% confidence intervals (CI) are shown per 1 standard deviation difference in the metabolic measures and adjusted for sex, age, and BMI. FDR, false discovery rate.

Table S1. Insulin sensitivity and secretion in participants with low and high visceral adipose tissue mass.

	N (Low/High VAT)	Low VAT	High VAT	P-value
VAT mass (kg)	87/87	1.55 (1.04; 1.92)	3.77 (2.93; 4.58)	
Insulin sensitivity at fasting				
HOMA-IR	87/87	1.26 (0.85; 1.63)	1.96 (1.39; 3.15)	1.7E-4
Adipo-IR (pmol/l*mmol/l)	84/85	16.3 (9.8;27.4)	29.4 (18.9; 45.1)	0.010
Insulin sensitivity and secretion during OGTT				
$Insulin_{AUC0\text{-}30min}/glucose_{AUC0\text{-}30min}$ (pmol/mmol)	54/32	14.6 (12.5; 25.8)	23.6 (15.4; 36.3)	0.085
$Insulin_{AUC0\text{-}120min}/glucose_{AUC0\text{-}120min}$ (pmol/mmol)	63/44	25.6 (18.9; 34.4)	30.0 (24.2; 48.5)	0.312
Matsuda ISI	65/44	25.7 (19.2; 39.2)	19.1 (10.6; 28.5)	0.045
Insulin sensitivity during clamp				
Adipo-IR (pmol/l*mmol/l)	59/59	14.5 (11.3; 23.2)	31.8 (17.7; 44.0)	0.017

LL = low liver lipids (≤1.85%); MEL = mildly elevated liver lipids (>1.85% and ≤5.56%); Adipo-IR = adipose tissue insulin resistance index; ISI = insulin sensitivity index; VAT = visceral adipose tissue. Data represented as median (1st quartile; 3rd quartile). The comparisons between low and high VAT are adjusted by age, sex, and BMI using multiple linear regression analysis. The P-value refers to testing the effect of VAT grouping adjusted for age, sex, and BMI.
