# Supplementary material for: Mildly elevated liver lipid content is characterised by reduced insulin sensitivity
Source: JHEP Rep. 2025 Aug 6;7(11):101535. doi: 10.1016/j.jhepr.2025.101535 (PMC12529492; doi:10.1016/j.jhepr.2025.101535)
Supplement: Multimedia component 3 [file mmc3.docx]

**JHEP Reports**

**CTAT methods**

Tables for a “Complete, Transparent, Accurate and Timely account” (CTAT) are now mandatory for all revised submissions. The aim is to enhance the reproducibility of methods.

- Only include the parts relevant to your study
- Refer to the CTAT in the main text as ‘Supplementary CTAT Table’
- Do not add subheadings
- Add as many rows as needed to include all information
- Only include one item per row

**If the CTAT form is not relevant to your study, please outline the reasons why:**

|  |
| --- |

- 1. **Antibodies**

| **Name** | **Citation** | **Supplier** | **Cat no.** | **Clone no.** |
| --- | --- | --- | --- | --- |
| **not applicable** |  |  |  |  |

- 1. **Cell lines**

| **Name** | **Citation** | **Supplier** | **Cat no.** | **Passage no.** | **Authentication test method** |
| --- | --- | --- | --- | --- | --- |
| **not applicable** |  |  |  |  |  |

- 1. **Organisms**

| **Name** | **Citation** | **Supplier** | **Strain** | **Sex** | **Age** | **Overall n number** |
| --- | --- | --- | --- | --- | --- | --- |
| **Homo sapiens** | **-** | **-** | **-** | **62 men/140 women** | **median 48yr (IQR 41, 59)** | **202** |

- 1. **Sequence based reagents**

| **Name** | **Sequence** | **Supplier** |
| --- | --- | --- |
| **not applicable** |  |  |

- 1. **Biological samples**

| **Description** | **Source** | **Identifier** |
| --- | --- | --- |
| **Fasting serum** | **Study participants** | **-** |

- 1. **Deposited data**

| **Name of repository** | **Identifier** | **Link** |
| --- | --- | --- |
| **not applicable** |  |  |

- 1. **Software**

| **Software name** | **Manufacturer** | **Version** |
| --- | --- | --- |
| **Carimas** | **Turku PET Centre** | **1.0-2.10** |
| **IBM SPSS** | **IBM** | **27** |
| **R** | **The R Foundation** | **4.3.3 and 4.5.0** |

- 1. **Other (*e.g*. drugs, proteins, vectors etc.)**

| **not applicable** |  |  |
| --- | --- | --- |
|  |  |  |

- 1. **Please provide the details of the corresponding methods author for the manuscript:**

| **Miikka-Juhani Honka, University of Turku, Turku PET Centre, Kiinamyllynkatu 4-8, 20520 Turku, Finland,** **tel: +35823132798,** **Fax: +358 2 231 8191, mjhonk@utu.fi** |
| --- |

**2.0 Please confirm for randomised controlled trials all versions of the clinical protocol are included in the submission. These will be published online as supplementary information.**

| **not applicable** |
| --- |
